# Supplementary material for: Reduction of bioburden on large area surfaces through use of a supplemental residual antimicrobial paint
Source: PLoS One. 2024 Sep 6;19(9):e0308306. doi: 10.1371/journal.pone.0308306 (PMC11379219; doi:10.1371/journal.pone.0308306)
Supplement: Attachment — Submitted filename: Response to Reviewers.docx [file pone.0308306.s002.docx]

We thank the editor and reviewers for their kind comments. Please find below our line-by-line responses (in italics) to the comments from the editor and reviewers.

**Editor’s comments**

1. Please ensure that your manuscript meets PLOS ONE's style requirements, including those for file naming. The PLOS ONE style templates can be found at 
<https://journals.plos.org/plosone/s/file?id=wjVg/PLOSOne_formatting_sample_main_body.pdf> and 
<https://journals.plos.org/plosone/s/file?id=ba62/PLOSOne_formatting_sample_title_authors_affiliations.pdf>

*The manuscript was reviewed to ensure that it meets PLOS ONE style requirements.*

2. Thank you for stating the following in the Competing Interests section: 

"Conflicts of interest: Jennifer Hiras, Jackie L. Kurzejewski, Alexander E. McInroy, Anthony G. Frutos, Mark R. Langille, and Joydeep Lahiri are employees at Corning Incorporated; Corning has commercialized the copper-glass ceramic additive (Corning® Guardiant®) used in the antimicrobial paint reported. This study was funded by Corning Incorporated and the University of Arizona received fees for services rendered for the processing and assay of the collected samples for the detection and quantification of HPC bacteria, coliforms, and Escherichia coli."
 
We note that one or more of the authors are employed by a commercial company: Corning Incorporated

A. Please provide an amended Funding Statement declaring this commercial affiliation, as well as a statement regarding the Role of Funders in your study. If the funding organization did not play a role in the study design, data collection and analysis, decision to publish, or preparation of the manuscript and only provided financial support in the form of authors' salaries and/or research materials, please review your statements relating to the author contributions, and ensure you have specifically and accurately indicated the role(s) that these authors had in your study. You can update author roles in the Author Contributions section of the online submission form.

*Author roles have been reviewed and updated.*

Please also include the following statement within your amended Funding Statement. 
“The funder provided support in the form of salaries for authors [insert relevant initials], but did not have any additional role in the study design, data collection and analysis, decision to publish, or preparation of the manuscript. The specific roles of these authors are articulated in the ‘author contributions’ section.”
If your commercial affiliation did play a role in your study, please state and explain this role within your updated Funding Statement.

*An amended Funding Statement has been provided at the end of this document.*

B. Please also provide an updated Competing Interests Statement declaring this commercial affiliation along with any other relevant declarations relating to employment, consultancy, patents, products in development, or marketed products, etc.

Within your Competing Interests Statement, please confirm that this commercial affiliation does not alter your adherence to all PLOS ONE policies on sharing data and materials by including the following statement: ""This does not alter our adherence to  PLOS ONE policies on sharing data and materials.” (as detailed online in our guide for authors <http://journals.plos.org/plosone/s/competing-interests>) . If this adherence statement is not accurate and  there are restrictions on sharing of data and/or materials, please state these. Please note that we cannot proceed with consideration of your article until this information has been declared.

Please include both an updated Funding Statement and Competing Interests Statement in your cover letter. We will change the online submission form on your behalf.

*An amended Funding Statement and Competing Interests Statement have been provided at the end of this document.*

3. Please include captions for your Supporting Information files at the end of your manuscript, and update any in-text citations to match accordingly. Please see our Supporting Information guidelines for more information: <http://journals.plos.org/plosone/s/supporting-information>.

*A “Supporting Information” section was added at the end of the manuscript including a caption for the supporting information file.*

4. Please review your reference list to ensure that it is complete and correct. If you have cited papers that have been retracted, please include the rationale for doing so in the manuscript text, or remove these references and replace them with relevant current references. Any changes to the reference list should be mentioned in the rebuttal letter that accompanies your revised manuscript. If you need to cite a retracted article, indicate the article’s retracted status in the References list and also include a citation and full reference for the retraction notice.

*The reference list has been reviewed to ensure it is complete and correct.*


**Reviewers' comments:**

Reviewer's Responses to Questions
**Comments to the Author**

1. Is the manuscript technically sound, and do the data support the conclusions?

The manuscript must describe a technically sound piece of scientific research with data that supports the conclusions. Experiments must have been conducted rigorously, with appropriate controls, replication, and sample sizes. The conclusions must be drawn appropriately based on the data presented.
Reviewer #1: Yes
Reviewer #2: Yes

 2. Has the statistical analysis been performed appropriately and rigorously?
Reviewer #1: Yes
Reviewer #2: Yes

 3. Have the authors made all data underlying the findings in their manuscript fully available?

The [PLOS Data policy](http://www.plosone.org/static/policies.action#sharing) requires authors to make all data underlying the findings described in their manuscript fully available without restriction, with rare exception (please refer to the Data Availability Statement in the manuscript PDF file). The data should be provided as part of the manuscript or its supporting information, or deposited to a public repository. For example, in addition to summary statistics, the data points behind means, medians and variance measures should be available. If there are restrictions on publicly sharing data—e.g. participant privacy or use of data from a third party—those must be specified.
Reviewer #1: Yes
Reviewer #2: Yes

 4. Is the manuscript presented in an intelligible fashion and written in standard English?

PLOS ONE does not copyedit accepted manuscripts, so the language in submitted articles must be clear, correct, and unambiguous. Any typographical or grammatical errors should be corrected at revision, so please note any specific errors here.
Reviewer #1: Yes
Reviewer #2: Yes

 5. Review Comments to the Author

Please use the space provided to explain your answers to the questions above. You may also include additional comments for the author, including concerns about dual publication, research ethics, or publication ethics. (Please upload your review as an attachment if it exceeds 20,000 characters)
Reviewer #1: Dear authors,

Thank you for this interesting manuscript. I have just some comments:
Line 71: Please delete "CFU" (mentioned in the next line again):

”*CFU” was deleted.*

Line 103/104: Why is the control paint used for 24 lockers in women's locker room and the antimicrobial paint for 41 lockers?

*We chose to use similar numbers of controls for the men’s and women’s locker rooms (25 and 24 lockers, respectively). Due to the larger number of lockers in the women’s locker room relative to the men’s locker room, this left 27 and 41 lockers, respectively, for the antimicrobial paint in the men’s and women’s locker rooms.*

Line 163: Please defined "RLU" (first occurrence):

*The following was added to define RLU: “relative light unit (RLU)”.*

Reviewer #2: The article provides a comprehensive description of the findings of the study. The methodology employed is thoroughly explained. The results are presented in a clear and concise manner, and are supported by relevant data. The discussion section of the article presents a well-informed analysis of the results, drawing on relevant literature and theoretical frameworks.

**Updated Funding Statement**

Corning Incorporated provided support in the form of salaries for authors [JH, JK, AM, ML, AF, JL], but did not have any additional role in the study design, data collection and analysis, decision to publish, or preparation of the manuscript. The specific roles of these authors are articulated in the “author contributions” section. The University of Arizona received fees for services rendered for the processing and assay of the collected samples for the detection and quantification of HPC bacteria, coliforms, and Escherichia coli.

**Updated Competing Interests Statement**

The authors [JH, JK, AM, ML, AF, JL] are employees of Corning Incorporated. This does not alter our adherence to PLOS ONE policies on sharing data and materials. Corning Incorporated has commercialized the copper-glass ceramic additive (Corning® Guardiant®) used in the antimicrobial paint reported.
